# Major Depression and Multiple Sclerosis - A Case Report

**Published:** 2013-09-25

**Authors:** S Moore

**Affiliations:** Tulane University School of Medicine, Department of Psychiatry

**Keywords:** major depression, multiple sclerosis, disabling disease, non-traumatic disability

## Abstract

Multiple Sclerosis (MS) is a chronic and disabling disease with a considerable social impact and economic consequences. In Europe, it is the most common cause of non-traumatic disability in young adults. Existing therapies that target immune modulation are largely ineffective in halting the progression of the disease and are fraught with severe side effects. Therefore, managing the comorbidities of MS is of utmost importance for long-term patient care and quality of life.

## Introduction

Multiple Sclerosis (MS) is a chronic and disabling disease with considerable social impact and economic consequences. In Europe, it is the most common cause of non-traumatic disability in young adults [**1**]. Existing therapies that target immune modulation are largely ineffective in halting the progression of the disease and are fraught with severe side effects. Therefore, managing the comorbidities of MS is of utmost importance for long-term patient care and quality of life. 

Major Depression is the most common neuropsychiatric comorbidity of MS, with an approximate 50% lifetime prevalence rate [**2**]. As with other chronic illnesses, depression has a profound effect on quality of life, medication adherence, and progression to disability [**3**]. The following case report illustrates the unique challenges of treating depression in MS and underscores the need for collaborative neuropsychiatric care for this population.

## Case report

HPI: A 44-year-old African-American Male with secondary progressive MS and no past history of mental illness was referred to the Veteran’s Administration Outpatient Psychiatry clinic following persistent complaints of insomnia and depression to other providers. The patient had been diagnosed with MS seven years prior and had poor response to numerous interferon therapies. 

 A review of the medical record showed that physicians in primary care, neurology, and physical rehabilitation all documented the patient's depressed affect, poor sleep, and rumination on the lack of a cure for MS. Various antidepressants and hypnotics had brought no improvement for the patient’s mood. By the time of the initial Psychiatric evaluation, the patient’s disease had progressed to the point where he was wheelchair bound and living in a nursing home. His primary depressive symptoms were anhedonia, decreased concentration, increased guilt, and hopelessness. He also mentioned feeling isolated and abandoned by family. Again, he insisted that only death or a cure for MS would relieve his depression and seemed to have little recognition that the disease would be ultimately fatal. Otherwise, he denied any pain, psychotic symptoms, anxiety, or history of substance abuse. 

 Laboratory Studies and Physical Exam: MRI showed prominence of lateral ventricles and generalized atrophy, along with extensive white matter changes in bilateral cerebral hemispheres, pons, cerebellum, and medulla. Physical exam was notable for a right gaze preference, horizontal nystagmus, slurred speech and markedly decreased strength (2/5) and tone on the right side. MMSE 29/30

 Medications: Duloxetine 30mg qam / 60mg qhs, alprazolam 1mg qhs, amitriptyline 25mg qhs, trazodone 200mg qhs, baclofen 20mg BID, Interferon Beta-1A 22mcg subq 3x/week, quinapril 20mg BID, metoprolol 25mg BID, and lactulose 10mg/15ml BID

 Clinical Course: First, in order to decrease the contribution of polypharmacy to his medication regimen, amitriptyline and trazodone were discontinued. Because the patient had failed multiple antidepressants, the treatment plan focused on a course of supportive-expressive therapy in order to process the meaning of his diagnosis, build a therapeutic relationship, and bolster defense mechanisms. The therapy consisted of six 30-minute sessions where the patient was encouraged to explore aspects of personal identity that MS could not change, and what he hoped to accomplish with the rest of his life. Though his insomnia did not respond to increases in alprazolam nor a trial of zolpidem, he continued to attend therapy regularly and found solace in rediscovering spirituality and writing music. The patient insisted that this was the first encounter in which a doctor directly confronted issues surrounding death and dying and expressed great gratitude for the honesty of the exchange. Later, nurses at his facility also remarked on his brighter affect and greater participation in self-care. 

## Discussion

Clinicians who care for patients with MS should expect to encounter depressive affective disorders at rates higher than those in most other chronic medical disorders and need to screen accordingly [**3**]. Scales such as the Endicott criteria for the depression in the medically ill can assist in clarifying the diagnosis but there is no substitute for a clinical interview. Diagnosing MD in the context of MS is further complicated by the overlap of symptoms such as fatigue, poor concentration, poor sleep, and appetite disturbances. Though this particular patient had reached an advanced disease state, there is no evidence that the severity of neurologic MS symptoms correlate with the severity of depression [**4**]. However, severity of depression in MS has been correlated with loss of volume in frontal lobe on MRI, as it was seen in this patient. 

 Depression in MS might also have a different etiology from depression in a neurologically intact population, which is likely related to decreased brain volume and disturbances in the HPA axis [**5**]. Cognitively impaired MS patients are more likely to adopt a maladaptive approach characterized by high levels of avoidance when dealing with problems generated by their disability [**6**]. In this case presentation immature psychological coping mechanisms such as denial and repression were very prominent. While there are relatively few trials of antidepressant medication in MS- and only two have been declared high quality- the results have consistently shown a greater improvement in symptoms (with both SSRI and TCA) over placebo [**5**]. Furthermore, trials that use talk therapies such as CBT (cognitive behavioral therapy) in MS have shown just as effective as medication in treating depression, and combining the two provides a greater effect than either alone. As such, a consensus statement by the National MS Society in the USA declared that the gold standard of treatment is an individualized biopsychosocial approach that combines psychotherapy and medication [**3**]. As illustrated in the case, working collaboratively with other physicians can be difficult even in a system in which multiple specialists are available. Though literature clearly supports the integration of psychotherapy in depression treatment, its use in clinical practice is relatively rare. As the burden of depression on health care outcomes continues to grow, familiarity with as many multiple treatment modalities as possible is essential.
